# Rethinking mental health wellness among adolescents living with HIV in the African context: An integrative review of mental wellness components

**DOI:** 10.3389/fpsyg.2022.955869

**Published:** 2022-09-20

**Authors:** Zaida Orth, Brian Van Wyk

**Affiliations:** School of Public Health, Faculty of Community and Health Sciences, University of the Western Cape, Bellville, South Africa

**Keywords:** mental wellness, adolescents living with HIV, integrative review, mental health, Africa

## Abstract

**Objective:**

Adolescents living with HIV (ALHIV) are considered to be at heightened risk for developing mental health problems in comparison to their peers due to the burden of living with a stigmatized condition and managing a chronic condition. Poorer mental health outcomes among ALHIV are associated with lower rates of adherence to anti-retroviral therapy (ART). It is necessary to improve mental wellness among ALHIV as this acts as a buffer against developing mental health problems which, if left untreated can evolve into mental health disorders. Research on mental wellness concepts among ALHIV is underdeveloped which is associated with a lack of appropriate measures of mental wellness. We conducted an integrative review to conceptualize mental wellness and consider the critical components for measuring mental wellness in ALHIV.

**Method:**

An integrative review of published literature focusing on mental wellness of ALHIV in the African context was conducted. The process was guided by the PRISMA operational steps. As part of our problem identification phase, we drew on findings from a previous systematic review of mental wellness instruments and a qualitative photovoice study on exploring the experiences of ALHIV, to develop an initial framework of 13 mental wellness concepts and behaviors which informed the search strategy.

**Results:**

The review included 17 articles from which we identified six mental wellness concepts: Connectedness, Sense of Coherence (SOC), Self-esteem, Self-acceptance, Hope for the Future and Spirituality as well as six behaviors facilitating mental wellness: Coping, Resilience, Purpose in Life (goals), Self-efficacy, Adherence Self-efficacy, and Leisure Activities. All of these concepts and behaviors have been noted in our previous research (systematic review and qualitative work), with the exception of adherence self-efficacy. Based on the findings from this review and our previous work, we adapted the Salutogenic Model of Health developed by Antonovsky in 1987, to propose a Salutogenic Model of Mental Wellness (SMoMW) for ALHIV in the African context. This SMoMW may be used to develop an age and culturally appropriate measure of mental wellness for ALHIV.

**Conclusion:**

The findings from this review used to conceptualize mental wellness among ALHIV which can be used to develop a measurement of mental wellness.

## Background

Mental health as an integral component of overall health and wellbeing, has received global acknowledgment, as evidenced in its inclusion in the Sustainable Development Goals (SDGs), the World Health Organization's (WHO) Mental Health Action Plan (2013–2030), WHO's mental health gap action programme (mhGAP) and the United Nations Children's Fund (UNICEF) development of the Measurement of Mental Health Among Adolescents at the Population Level (MMAP) (World Health Organization, 2013, 2020a; UNICEF, 2019). Adolescent mental health, in particular, is receiving more attention, because adolescence is a time of critical development that sets the course for mental health and wellness across the life course (World Health Organization, 2014; Bentley et al., 2019). UNICEF argues that since half of all mental disorders have their onset during adolescence, intervention during the adolescent years is essential to prevent the development of chronic mental illness conditions.

Further, WHO observes that 1 in 7 adolescents between the ages of 10–19 years, experience a mental health disorder (World Health Organization, 2020b). The prevalence of mental health disorders accounts for 13% of the global burden of disease among adolescents, with suicide being reported as the fourth leading cause of death among 15–19 years old (World Health Organization, 2020b). It is further reported that adolescents living with HIV (ALHIV) are at increased risk of experiencing ill mental health (compared to their peers) due to the double burden of living with a stigmatized infectious disease and a managing a life-long chronic condition (Vreeman et al., 2017; Woollett et al., 2017; Sherr et al., 2018; Laurenzi et al., 2020). Findings from a recent systematic review reported high prevalence rates of mental health problems among ALHIV, with 24–27% of participants scoring positive for having a psychiatric disorder and 30–50% showing symptoms of emotional and behavioral difficulties or significant psychological distress (Dessauvagie et al., 2020). Other research with ALHIV report high prevalence rates of symptoms of depression, anxiety, post-traumatic stress disorder (PTSD), internalized stigma, hopelessness, fear, or suicidality (Woollett et al., 2017; Sherr et al., 2018; West et al., 2019; Okumu et al., 2021; Nguyen et al., 2022). Further, it is shown that these poor mental health outcomes are associated with increased incidence of risky behaviors, sub-optimal adherence to antiretroviral therapy (ART) and low retention in care (RiC)—which, in turn, may lead to viral load rebound and virologic failure (Hudelson and Cluver, 2015; Chory et al., 2022; Nguyen et al., 2022).

WHO defines mental health as more than the absence of illness, but as “a state of wellbeing, in which an individual realizes his or her own abilities, can cope with the normal stresses of life, can work productively and is able to make a contribution to his or her community” (World Health Organization, 2019). However, in mental health research, mental illness has been predominantly used as a euphemism for (or indicator of) mental health. This formulation of mental health excludes mental wellness, which is critical to the prevention of mental illness (or disorders) and the over-all promotion of positive mental health (Keyes, 2005). In the case of adolescents living with a chronic condition (such as ALHIV) [positive] mental wellness is a critical buffer against developing mental health disorders and for self-management of their chronic condition.

To date, mental wellness concepts for adolescents, particularly ALHIV, are underdeveloped and lack robust measurement instruments to stimulate research on this topic. Studies on mental wellness have been done with adult populations and applied to adolescents (Keyes, 2005; Roscoe, 2009; Ahanonu and Jooste, 2016). While research has shown that there are some similarities between adolescents and adults, it should also be considered that adolescence is a unique developmental period that is characterized by rapid physiological and neurological growth and cognitive development (UNAIDS, 2016; Lake et al., 2019), that occurs within the social context of various transitions to adulthood. Therefore, more research is necessary to explore what mental wellness means for adolescents, especially those living with a chronic condition like HIV, to develop culturally and age-appropriate instruments that can be used to monitor and evaluate progress on improving their overall mental wellbeing. This review aims to conceptualize mental wellness among ALHIV in the African context and consider the critical components for measuring mental wellness [state and behavior] in ALHIV (Orth and van Wyk, 2022). This integrative review forms part of a multi-phase study aimed at developing an instrument to measure mental wellness among ALHIV.

## Methods

**Figure 1:**
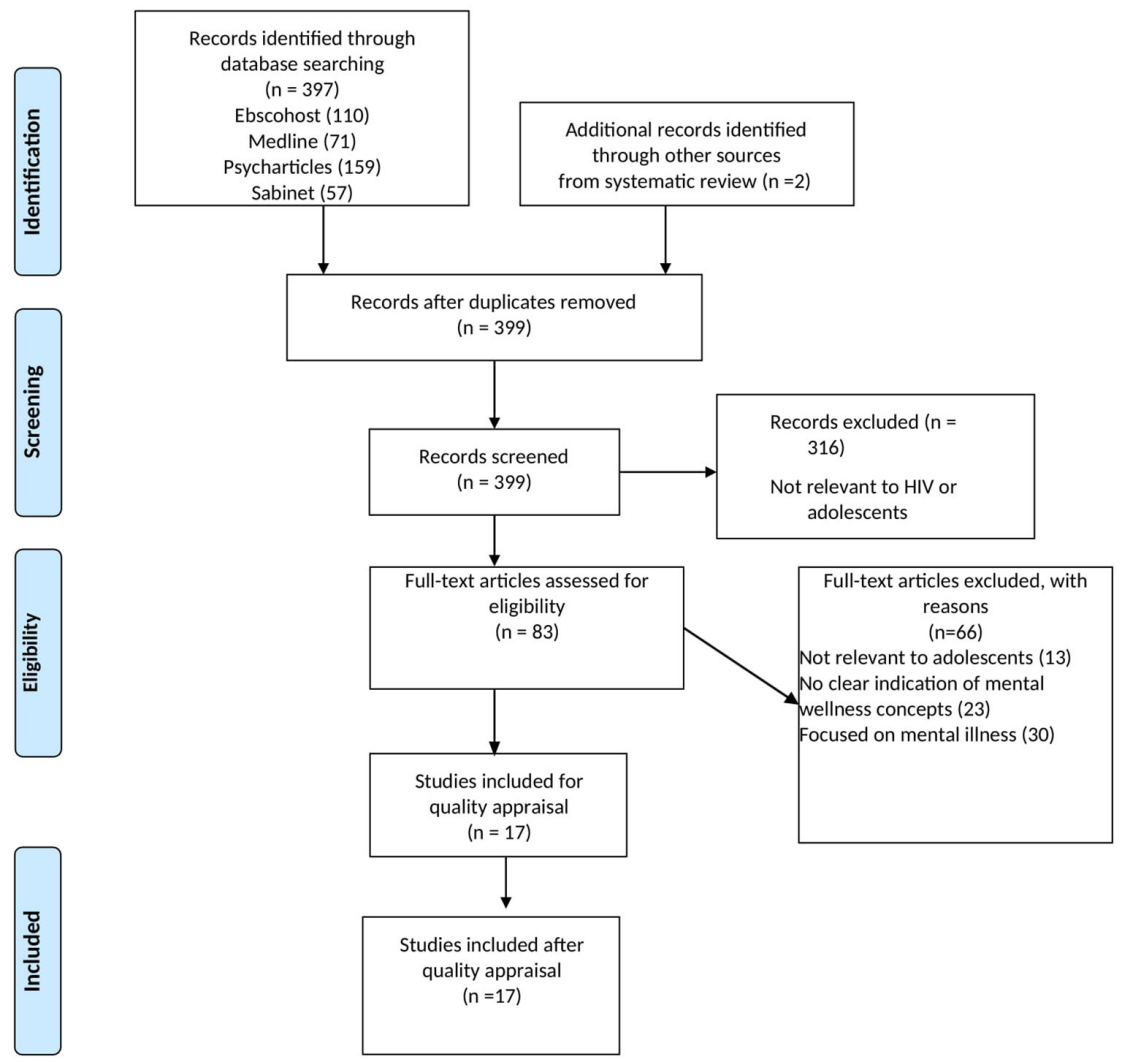
PRISMA flow diagram of integrative review (Page et al., 2021).

The methods for this review have been described in detail in the protocol (Orth and van Wyk, 2022). The integrative review has been identified as a unique tool in healthcare for synthesizing theoretical and empirical evidence investigations available on a given topic or phenomena to provide a more comprehensive understanding of a certain healthcare problem or other phenomenon. To accomplish this, a range of methodologies may be utilized to fully capture the context, processes, and subjective elements of the topic under investigation (Souza and De, 2010). Therefore, integrative reviews can contribute to theory development and have practical applicability to informing policy and programmes (Whittemore and Knafl, 2005). The existing body of literature on mental health among adolescents is varied and complex as there are many concepts associated with mental health research ranging from positive aspects such as “resilience” and “self-efficacy” to negative aspects such as “depression” and “anxiety”. As such, it is not possible for one study to capture all the dimensions associated with mental health. To provide a complete picture of the available literature and to fully investigate the concept of mental wellness among ALHIV, we followed the integrative review steps proposed by Whittemore and Knafl (2005): (1) problem identification; (2) literature search; (3) data evaluation; (4) data analysis: and (5) presentation of the integrative review guided by the Preferred Reporting Items for Systematic Review and Meta-Analyses (PRISMA) guidelines (Figure 1) (Page et al., 2021).

### Problem identification

As described in the protocol (Orth and van Wyk, 2022) the problem identification is a crucial step in an integrative review, and as such, this was treated as a phase in itself. From our initial reading of the literature, we have identified two recurring issues: firstly, there is a lack of validated mental health instruments for adolescents; and secondly, despite a growing body of research, the question of how mental wellness should be defined remains largely unresolved (Roscoe, 2009; Manderscheid et al., 2010; Manwell et al., 2015). To investigate this, we have proposed to follow two research questions to aid problem identification:

1) How is the concept of mental wellness defined in research involving adolescents?
2) What indicators of mental wellness are being explored/investigated in research?

To answer the abovementioned questions, we conducted a systematic review of all instruments used to measure mental wellness in adolescents (Orth et al., 2022). The sub-analysis of instruments used among adolescents living with a chronic condition (Orth and van Wyk, 2021a) revealed that Health-Related Quality of Life (HRQoL) instruments were frequently used to measure physical and psychosocial wellbeing among adolescents living with a physical chronic condition. However, these HRQoL instruments often include mental illness and mental wellness indicators which raises the question—to what degree is the absence of mental disorder symptoms equal to a high degree of mental wellness? As such, we argued that more instruments need to be developed in LMICs to give insight into which constructs of mental wellness are important to improving overall mental wellbeing for adolescents living in these contexts (Orth and van Wyk, 2021a; Orth et al., 2022). Though there was a lack of clear definition of mental health, we identified 13 mental wellness concepts from 79 instruments, namely: life satisfaction, mental wellbeing [general], resilience, self-efficacy, self-esteem, connectedness, coping, self- control, mindfulness/spiritual, hope, sense of coherence, happiness, and life purpose (Orth et al., 2022).

To explore the relevance of these 13 concepts for ALHIV, we conducted a second order analysis of qualitative data that emanated from a photovoice study exploring the experiences of ALHIV receiving ART at three public healthcare facilities in the Western Cape metropole of South Africa (Orth and van Wyk, 2021b). The photovoice technique allowed participants to lead the narrative and express themselves creatively through taking photographs with cell phone cameras (Orth and van Wyk, 2021b). As they spoke about their experiences, discussions around what mental wellness means to them emerged naturally. Through discourse analysis we identified six themes that depicted mental wellness concepts that were prominent in their experiences, namely: connectedness, spirituality and mindfulness, social coherence and awareness, self-esteem, self-acceptance, and sense of coherence. In addition, the adolescents gave accounts of six behaviors facilitating mental wellness namely: self-efficacy, coping, resilience, life purpose, engagement in enjoyable life activities and physical functioning (Orth and van Wyk, 2021b). The findings from the systematic review and photovoice study provided us with an initial framework of concepts and behaviors that informed our understanding of potential domains to include in developing a mental wellness instrument. However, as we explored each of these concepts further, we noted that traditional definitions of several of the concepts were not clearly delimited and overlapped with one another. For example, in the literature concepts like “self-esteem”, “self-worth” and “self-acceptance” are often not clearly distinguished from one another. To address this problem, we used the mental wellness concepts and behaviors identified in the systematic review and photovoice study and developed a search strategy for the integrative review to further investigate the meaning of these to aid in the conceptualization of mental wellness among ALHIV.

### Literature search

We systematically searched the following databases: Ebscohost (Psycharticles, Academic Search Premier, SocIndex), Educational Resource Information Center (ERIC), Medical Literature Analysis Retrieval System Online (MEDLINE) and Sabinet. We performed multiple searches using each of the identified concepts: (connectedness OR social support OR belonging), (cope^*^ OR coping^*^), (hope), (purpose in life OR meaning in life OR sense of purpose), (physical functioning OR physical wellbeing), (resilience), (self-acceptance), (self-efficacy), (self-esteem), (sense of coherence), (spirit^*^ OR mindful^*^) AND (adolescen^*^ OR teenage^*^ OR young people OR youth) [AND] (HIV OR living with HIV). The search was completed at the end of April 2022 and included all studies published up until that period.

### Inclusion and exclusion criteria

The integrative review allows for an iterative process. Based on the findings from the previous studies, we adjusted the search from our original protocol, which aimed to include all adolescent populations to only focus on ALHIV (Orth and van Wyk, 2022). The purpose of this review is to identify mental wellness concepts that are significant to older ALHIV (aged 15–19 years), with the aim of developing an instrument that can be used to measure mental wellness of ALHIV living in South Africa. Our study selection was guided by the Population, Intervention, Comparison, Outcome and Time (PICOT) criteria (see Table 1).

**Table 1:** PICOT based inclusion criteria for literature review.

Patient population	Adolescents living with HIV in the African context
Intervention or interest	Definition or explanation of the identified mental wellness concepts and behavior
Comparison	Not applicable
Outcomes	Mental wellness, psychological wellbeing, positive mental health
Time	Any time

#### Inclusion criteria

- ALHIV (perinatally and behaviorally acquired) aged 10-19 years
- Studies based in the African context
- Clear focus on the identified mental wellness concept
- Qualitative, quantitative or mixed-methods studies

#### Exclusion criteria

- Studies focusing on or including mental illness as a concept
- Studies that were not peer reviewed

### Data evaluation

The screening and reporting of the review followed the Preferred Reporting Items for Systematic Reviews and Meta-Analyses (PRISMA) guideline and checklist (Figure 1). The number of hits for each search was recorded and exported to Endnote for review. The Mixed Methods Appraisal Tool (MMAT) was used to assess the methodological quality of the studies as it allows for summarizing the overall quality across a range of study designs (Hong et al., 2018). This allowed us to ensure that all the included studies were of good quality. After finalizing the selection of included articles, we extracted the data into an excel sheet focusing on (1) Bibliometric data (authors, title, year, country), (2) population group (age and sample size), (3) study design (type of study, methods) and (4) outcome of interest (mental wellness concepts defined or measured in the article).

### Data analysis

We extracted the relevant data and organized it in an excel sheet to prepare for the analysis. The data was analyzed using a narrative framework analysis for qualitative and quantitative studies. Framework analysis involves engaging in a systematic process of data familiarization and identifying a thematic framework to chart the data (Snilstveit et al., 2012). Depending on the study and phenomena under investigation, an initial framework can either be borrowed from previous studies or can be developed from key concepts (Whittemore and Knafl, 2005; Snilstveit et al., 2012). As mentioned, our analytical framework was developed by using the mental wellness concepts and behaviors identified from the systematic review (Orth et al., 2022) and photovoice data (Orth and van Wyk, 2021b). We then applied the analytical framework by comparing and indexing the mental wellness concepts that were extracted from articles in the integrative review with the concepts in our framework (i.e., connectedness, coping, hope, purpose in life, physical functioning, resilience, self-acceptance, self-efficacy, self-esteem, sense of coherence, spirituality). Emerging concepts that were not represented in the initial framework were subsequently added to the list. Once all the concepts were presented in the updated analytical framework, we charted the data by defining each concept (as defined in the included studies) (Table 2).

**Table 2:** Mental wellness concepts and behaviors from the review.

**Mental wellness concept/behavior**	**Definition or interpretation**	**Quotes from included studies**	**References**
Connectedness	Sense that one has satisfying relationships with others, believing that one is cared for, loved, esteemed, and valued, and providing friendship or support to others	“It seems the combination of disclosure and social support gave the adolescents a unique group feeling, a feeling of belonging, which seemed to be some of the key factors in their development of self-esteem and coping with HIV”	Adegoke and Steyn, 2018; Dow et al., 2018; Govindasamy et al., 2020; Kimera et al., 2020; Gitahi et al., 2021
		“In another study examining the benefits of family and social relationships for health and mental health of PLWH, family functioning significantly contributed to ART adherence and quality of life. Thus, strengthening positive family support and minimizing negative family interactions are crucial for increasing adherence rates”	Petersen et al., 2010; Midtbø, 2012; Mburu et al., 2014; Nöstlinger et al., 2015; Nabunya et al., 2020
		“Along with family members, peers who were also living with HIV featured prominently as a source of psychosocial support and friendship. Adolescents reported that through such peer connections, they could share coping strategies, make each other feel valued and offer each other a sense of identity”	Shabalala et al., 2016; Zanoni et al., 2019; Rencken et al., 2021
Coping	Coping refers to cognitive and behavioral efforts to manage (master, reduce, or tolerate) a troubled person- environment relationship	“At the interpersonal level, family and peer support emerged as key to assisting adolescents to cope”	Petersen et al., 2010; Midtbø, 2012; Mburu et al., 2014; Woollett et al., 2017; Adegoke and Steyn, 2018; Dow et al., 2018
		“Also at the individual level, a couple of adolescent respondents indicated how positive thinking and having goals for the future helped them to cope and suggested that instilling these in other children may be useful”	
Self-acceptance	A positive attitude toward yourself; acknowledge and accept multiple aspects of yourself including both good and bad qualities; and feel positive about your past life.	“It has been suggested that peer support group therapy for HIV positive adolescents positively affects their acceptance and perception of their disease”	Mburu et al., 2014; Bernays et al., 2015; Lentoor et al., 2016; Woollett et al., 2016; Zanoni et al., 2019
		“Although some adolescents reported that internalized HIV stigma had affected their ability to engage socially, many of these adolescents said that they were able to accept their situation eventually, regain their self-esteem, and interact with their families and peers, which in turn strengthened their self-efficacy and resilience.”	
		“Being self-assured and accepting oneself were the basis of this self-esteem”	
Resilience	The ability to mentally withstand or adapt to uncertainty, challenges, and adversity.	“The most salient theme to emerge from the study in relation to individual-level factors that might influence adolescents' experience of living with HIV was their resilience, sometimes tempered by internalized stigma”	Mburu et al., 2014; Woollett et al., 2016; Adegoke and Steyn, 2018; Zanoni et al., 2019; Kaunda-Khangamwa et al., 2020
		“Features of resilience in this group were underscored by beliefs and character traits that enabled their ability to manage their adversity, as well as social behaviors that created the agency necessary for success”	
Self-esteem	A person's overall subjective sense of personal worth or value	“They are able to talk about their health with other HIV positive adolescents and it is also suggested that peer support groups enable the adolescents to develop good self-esteem”	Mburu et al., 2014; Nöstlinger et al., 2015; Woollett et al., 2016; Gitahi et al., 2021
		“Some of them reported that knowing their status was a strength to them, one boy stating that “we have self-esteem because we know our status””	
		“By disclosing their status to peers, the adolescents in my study showed that they were empowered and, in a position, to take their own decisions regarding who to disclose to and where to seek support. This indicates self-esteem and confidence”	
Hope for the future	Emotion characterized by positive feelings about the immediate or long-term future.	“Considering the participants in my study, most of them were thriving and managed to remain positive, even though they knew they had HIV and had to be on ART for the rest of their lives.	Bernays et al., 2015; Adegoke and Steyn, 2018; Bakeera-Kitaka et al., 2020; Kimera et al., 2020
		Most of them had hopes and dreams for the future and had specific thoughts about what they wanted to do when they grew older”	
		“Hope was identified as an important motivation for protection. Many hoped that if they continued to adhere to their treatment, they would be able to live long enough to finish school, get a good job, get married, and have their own children. Some hoped that finally a cure for HIV might be found.”	
Spirituality	Psychological process of bringing one's attention to the internal and external experiences occurring in the present moment; concern for or sensitivity to things of the spirit or soul.	“Additionally, many reported that they trusted God and prayed for good health, wisdom, courage, strength and cure in future. As a result, they were more optimistic that all will be well with them in future”	Woollett et al., 2016; Adegoke and Steyn, 2018; Kimera et al., 2020
		“A strong theme emerging from adolescent participants was the idea that their own belief systems set the stage for their ability to be resilient. Many participants demonstrated a belief in fate with a comfort in the conviction that one is on the path one should be”	
Sense of Coherence	Degree of meaningfulness (motivational), comprehensibility (cognitive), and manageability (behavioral) that people feel in their life	“HIV positive adolescents, who thrive in spite of difficult challenges, can be said to have a strong SOC and resources at hand that enable them to cope with the challenges or stressors present in their lives. The knowledge of what these resources are can be used to promote SOC, leading to increased quality of life and wellbeing for this group of adolescents.”	Midtbø, 2012; Woollett et al., 2016
		“In other words, it can be said that disclosure was a main contributing GRR in enabling many of these adolescents develop and strengthen their SOC, which furthermore contributes to a movement toward health”	
Purpose in life (goals)	You have goals in life and a sense of directedness; feel there is meaning to your present and past life; hold beliefs that give life purpose; and have aims and objectives for living.	“Adolescents were motivated and had a sense of purpose. Some adolescents described carrying out an expanded range of duties, such as caring for their own children or younger siblings who were or were not living with HIV, with resilience, a deep sense of responsibility, hope for the future and optimism that eclipsed any sense of living with a chronic disease.”	Midtbø, 2012; Govindasamy et al., 2020
		“Importantly, these goals appeared to promote wellbeing by providing a sense of purpose and making them feel socially valued”	
Self-efficacy	A person's particular set of beliefs that determine how well one can execute a plan of action in prospective situations. Self- efficacy is a person's belief in their ability to succeed in a particular situation.	“Observable ART adherence levels depend on a range of factors, including self-efficacy i.e., the person's perception of their own ability to accomplish a behavioral task, which influences a person's development or maintenance of a health behavior at the affective, cognitive and motivational levels”	Mburu et al., 2014; Gitahi et al., 2021
		“In addition, many participants demonstrated self-reliance that was key to self-esteem: ‘If you don't believe in yourself, who will?”	
Leisure activities	Engaging and participating in activities that bring enjoyment	“Family, friends and leisure activities were also important positive factors that contributed to wellbeing”	Midtbø, 2012
		“Most of them also had leisure activities which they enjoyed, and some were very passionate about these activities, finding it a very important part of their lives”	
		“Leisure activities such as sports and drama were also activities that some of the participants were very passionate about. As mentioned, participation is connected to meaningfulness, which is the motivational component”	
Adherence Self- efficacy	Belief in one's ability to successfully adhere to treatment plans	“More specifically, adherence self-efficacy –defined as the confidence in one's ability to adhere to treatment plans, has been documented as an important predictor of medication adherence in the treatment of HIV and other medical conditions”	Gitahi et al., 2021

## Findings

The integrative review included 17 studies that focused on the mental wellness of ALHIV in the African context. As the review included both qualitative and quantitative studies, sample sizes ranged from 5 to 702. The majority of studies included in this review were conducted in South Africa (*n* = 5) and Uganda (*n* = 5). Most of the included studies used qualitative designs (*n* = 12) (Petersen et al., 2010; Midtbø, 2012; Mburu et al., 2014; Bernays et al., 2015; Shabalala et al., 2016; Woollett et al., 2016; Zanoni et al., 2019; Bakeera-Kitaka et al., 2020; Govindasamy et al., 2020; Kimera et al., 2020; Gitahi et al., 2021; Rencken et al., 2021); with only three quantitative [cross-sectional (Gitahi et al., 2021), longitudinal randomized clinical trial (Nabunya et al., 2020), secondary analysis (Nöstlinger et al., 2015)] and two mixed-methods study (Dow et al., 2018; Kaunda-Khangamwa et al., 2020). This may be indicative of a lack of mental wellness instruments for ALHIV, or it may reflect the research trends focusing on measuring the prevalence of mental health problems in ALHIV (Kidia et al., 2015; Vreeman et al., 2017; Laurenzi et al., 2020).

From the included studies we identified six mental wellness concepts: Connectedness, Sense of Coherence (SOC), Self-esteem, Self-acceptance, Hope for the Future and Spirituality as well as six behaviors facilitating mental wellness: Coping, Resilience, Purpose in Life (goals), Self- efficacy, Adherence Self-efficacy, and Leisure Activities (Table 2). All of these concepts and behaviors have been noted in our previous research (systematic review and qualitative work), with the exception of adherence self-efficacy. However, as our focus for this review was on ALHIV, this finding is not surprising. Studies have shown that adherence self-efficacy plays an important role in maintaining adherence—ALHIV (behaviorally or perinatally infected) are in a stage where they gradually experience greater responsibility—including learning to manage their health. Therefore, having a high sense of adherence self-efficacy can ease their transition to adult clinical care.

Based on our research, we argue that the above-mentioned concepts are significant indicators of mental wellness among ALHIV in the African context—however, Africa is a diverse continent and ALHIV are not a heterogenous group. For example, the study by Adegoke and Stein (Adegoke and Steyn, 2018) explore how resilience manifests among HIV positive adolescent Yoruba girls. The findings from the study demonstrate how the Yoruba culture may enable resilience (through and emphasis on family ties and social cohesion) or constrain it through gender relations that often perpetuate gender inequalities which put adolescent girls (especially those living with HIV at risk) (Adegoke and Steyn, 2018). As many of the included studies are qualitative, we argue that the development of the mental wellness measure will facilitate research investigating these concepts among ALHIV in the African context to better understand the role and influence of culture on mental wellness.

Table 2 provides a definition of each concept followed by quotes from the included studies to illustrate how the particular mental wellness concept or behavior is associated with improved mental wellness and/or physical health outcomes in ALHIV. Similar to the findings from our systematic review and qualitative work (Orth and van Wyk, 2021b; Orth et al., 2022), the quotes confirm that the mental wellness concepts and behaviors do not operate independently; rather these are interconnected and work collaboratively to promote mental wellness. For example, the study by Dow et al. (2016) focused on developing a mental health intervention for ALHIV in Tanzania to improve their resilience—to accomplish this, the intervention included resilience strategies to cope with stressful events such as enabling and supporting strong familial and social relationships, addressing stigma (using cognitive behavior therapy techniques to change negative thoughts to positive ones) and instilling hope for the future. According to Dow et al. the participants found the intervention to be highly acceptable and feasible and was associated with increased resilience among ALHIV. However, it is not clear from the study how mental health was measured—the authors' mention that participants were given a pre-intervention questionnaire which included mental health, but this is not reported. While the main aim of the mental health intervention was to improve resilience among ALHIV, the qualitative findings suggest that mechanisms used to trigger resilience, were associated with improvements in other mental wellness outcomes such as connectedness (familial and social relationships), self-esteem and self-acceptance (stigma reduction) and hope for the future. As such, it is critical to utilize measures that can capture a range of associated mental wellness outcomes. Without appropriate instruments, it is impossible to draw conclusions about the efficacy of interventions aimed at improving mental wellness. Measures that assess various mental wellness concepts/behaviors in parallel are useful, as these provide precise indications regarding the relationship (pathways) between these concepts and behaviors. Furthermore, such measures may provide information regarding the contribution of each of these mental wellness concepts and behaviors in heterogenous populations of ALHIV, and that can be critical in assessing interventions to improve treatment outcomes. This lends support for the need for integrated measures of mental wellness for ALHIV rather than instruments measuring single concepts/behaviors such as self-esteem for example.

### Toward a model of mental wellness

In this integrative review we unpacked the meaning of mental wellness for ALHIV, with the express aim of informing the development of an appropriate research instrument to measure mental wellness for ALHIV. Therefore, our approach moves away from traditional pathological inquiry of what causes mental illnesses to a salutogenic exploration of “what promotes mental wellness”. As such, we turn our attention to SOC which emerged as a key concept in our research and is considered to represent “the origins of health” from a salutogenic approach (Antonovsky, 1987; Mittelmark et al., 2015). SOC reflects the coping capacity of people to deal with everyday life stressors and consists of three elements: comprehensibility (cognitive—extent to which the problem/stressor understood), manageability (behavioral—perceived availability of resources and belief in ability to use successfully use them) and meaningfulness (motivational—extent that one wishes to cope) (Antonovsky, 1987; Mittelmark et al., 2015). SOC is a developmental concept which begins to form during adolescence and stabilizes by the age of 30 years (Antonovsky, 1987; Braun-Lewensohn et al., 2015; Mittelmark et al., 2015; Hochwälder, 2019; Carlén et al., 2020; Mjøsund, 2021). Therefore, from a life-course perspective, strengthening SOC as a health promoting factor may improve overall physical and mental wellness in later life.

**Figure 2:**
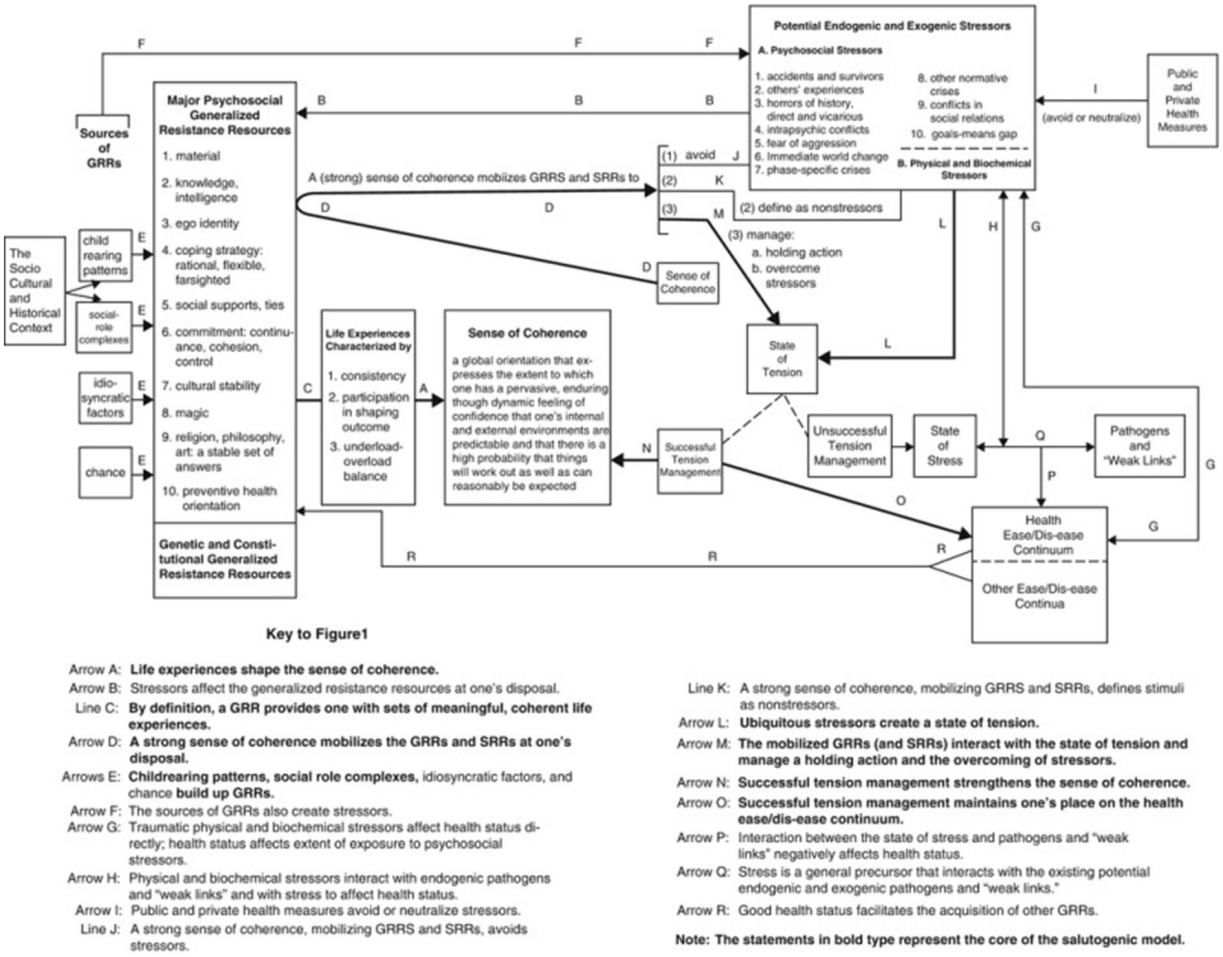
Antonovsky's salutogenic model of health (Mittelmark et al., 2015).

Antonovsky originally proposed SOC as the core concept of his Salutogenic Model of Health—which is aimed at explaining the origins of health and to describe how health can be promoted by focusing on wellness (Antonovsky, 1987; Braun-Lewensohn et al., 2015; Mittelmark et al., 2015; Hochwälder, 2019; Carlén et al., 2020; Mjøsund, 2021). The Salutogenic Model of Health was developed as an alternative to the pathological view of health/disease to improve health promotion by focusing on what makes people healthy. Unlike the pathological view, the salutogenic approach rejects the idea that homeostasis is a basic human condition; rather it argues that disease, illness, and decline are the norm (Antonovsky, 1987; Mittelmark et al., 2015). From this perspective, all individuals experience daily life stressors which causes immediate tension—however this tension may be resolved through effective coping and management strategies. Disease or illness occurs when the individual experiences long term stress resulting from their inability to resolve tension. As such, it is more constructive to focus on ways to improve an individual's adaptability to daily life stressors to promote overall health and wellness (Figure 2).

According to Antonovsky (Antonovsky, 1987; Mittelmark et al., 2015) the Salutogenic Model of Health represents a continuum model in which health is the result of continuous everyday life interactions between the individual, their experience of inevitable social, economic, cultural, psychosocial, and biological stressors, the availability of and access to health promoting resources and their capabilities in identifying and mobilize these resources to effectively overcome tensions resulting from stressors. Within this model, SOC reflects the individual capability to identify and mobilize resources, and the resources that promote health and facilitate coping with stressors are called Generalized Resistance Resources (GRRs) which can be genetic, material, constitutional and/or psychosocial resources (Antonovsky, 1987; Mittelmark et al., 2015). Based on this model, if an individual has a well-developed SOC and GRR, they are better able to identify SRRs and develop coping strategies to overcome specific challenges—for example a study by Polhuis et al. (2020) demonstrated how the Salutogenic Model of Health may be used to identify turning points and coping styles to help people with type 2 diabetes to adopt healthy eating habits.

**Figure 3:**
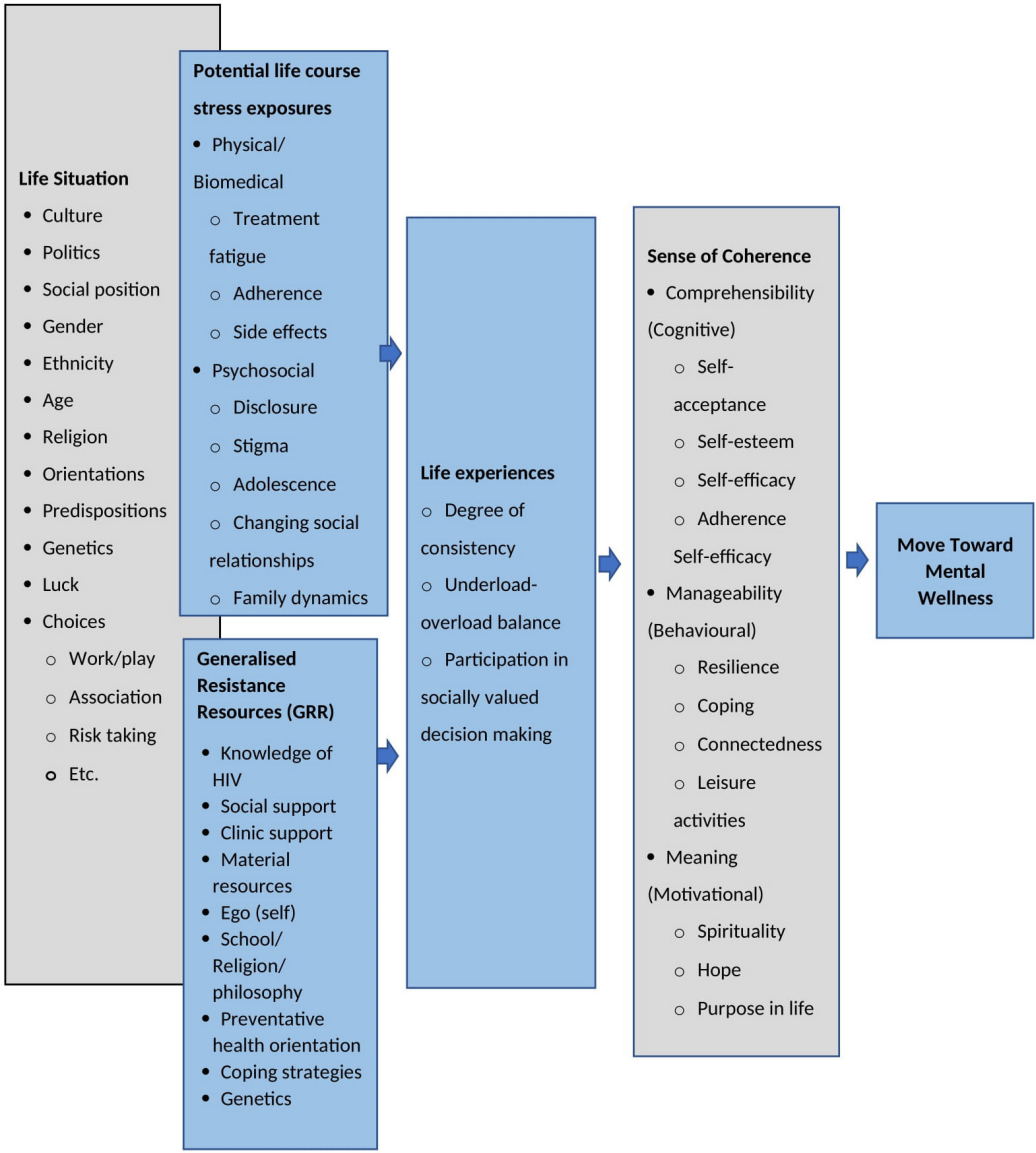
Salutogenic model of mental wellness for adolescents living with HIV adapted from Antonovsky, 1987 (Mittelmark et al., 2015) and Benz et al. (2014).

In reviewing SOC and the Salutogenic Model of Health, we found it to be useful in illustrating the relationships and associations between the mental wellness concepts and behaviors identified in this review. As such we propose an adapted Salutogenic Model of Mental Wellness (SMoMW) for ALHIV (Figure 3) may be used to develop an instrument to measure mental wellness among ALHIV (Benz et al., 2014; Mittelmark et al., 2015). Similar to the Salutogenic Model of Health, the SMoMW views mental wellness along a continuum which may be influenced by individual interaction with everyday life stressors. As the model emphasizes the role of context, it can be applied to adolescents in general or those living with a chronic condition like HIV. Within the SMoMW, the Life Situation represents the macro socio-cultural and historical context that shape individual lived experiences. This macro context may serve to build up or detract GRRs—for example, for ALHIV, HIV knowledge acquisition is considered to be an important resistance resource (GRR). However, age (life situation) plays an important role in knowledge HIV acquisition—older ALHIV who have been disclosed to may learn more about how to effectively manage adherence than younger adolescents who have not been disclosed to. As such, younger ALHIV may experience different daily stressors than older ALHIV due to their lack of knowledge which could negatively. On the other hand, stressors they experience from the lack of knowledge may be buffered by protective family relationships (connectedness) to help them manage their illness until they are old enough to be disclosed to. Similarly, as they grow older, adolescents are more likely to develop a stable Ego (self- acceptance, self-esteem etc) which may help them process the availability of GRRs and their ability to successfully utilize them (Antonovsky, 1987; Mittelmark et al., 2015). Additionally, ALHIV life situation may also exacerbate or ease their exposure to and experience of life stressors. For example, genetics may play a role in how an ALHIV experiences treatment fatigue or side effects (including neurological effects from long term use). Both the exposure to potential life course stressors and GRRs can influence to what extent an individual experiences life as coherent and meaningful. ALHIV who are able to utilize the GRRs available to them are more likely to experience consistency in their day to day lives (positive influence on adherence and development), balance responsibilities and leisure and be active participants in decisions which affect their lives. These life experiences shape SOC—which reflect the mental wellness concepts and behaviors identified in the review to promote mental wellness.

The SMoMW may be useful in developing a mental wellness measure for ALHIV. Not only does it emphasize the role of SOC and the associated concepts/behaviors as integral to mental wellness, but it also frames the dynamic interaction between SOC and health-promoting factors and stressors in relation to living with HIV over the life-course. Therefore, an instrument developed from the SMoMW may be beneficial as it would allow us to interrogate and explore the mental wellness needs of ALHIV as a heterogenous group with diverse demographic, social and clinical traits.

### Strengths and limitations

To our knowledge, this is the first review aimed at identifying and defining mental wellness concepts and behaviors that are relevant to ALHIV in the African context. The integrative review method has been critiqued for its potential for bias and lack of rigor (Whittemore and Knafl, 2005). However, a strength of this study is that the search strategy was developed through a rigorous process which involved a systematic review and a photovoice study which speaks to the validity of the concepts identified. However, we acknowledge that studies may have been omitted from the search due to access restrictions. Additionally, while we attempted to keep search terms as broad as possible, the lack of clear definitions of the included concepts may have resulted in articles being unintentionally omitted.

## Conclusion

The findings from the integrative review highlight the mental wellness concepts and behaviors which are significant to ALHIV in the African context. Based on the findings from this review, as well as our previous systematic review and qualitative work, we propose a Salutogenic Model of Mental Wellness for ALHIV that can be used to develop a mental wellness instrument. This instrument includes the following concepts: Connectedness, Self-esteem, Self-acceptance, Hope for the Future and Spirituality and behaviors: Coping, Resilience, Purpose in Life (goals), Self-efficacy, Adherence Self-efficacy, and Leisure Activities which are related to overall Sense of Coherence (SOC) to promote overall mental wellness. Such an instrument may be used to measure impact of interventions aimed at measuring mental wellness among ALHIV. Additionally, the instrument may provide much needed data on different mental wellness mechanism that can help us to better understand the relationship between different mental wellness concepts and behaviors, the significance for diverse groups of ALHIV and how these work together to influence and support adherence behaviors.

## Author contributions

ZO contributed to the conceptualization and management of the review process, fieldwork, data extraction, provided leadership and input to the review team at each stage of the project and the conceptualization, drafting, technical aspects, and critical revisions of the manuscript. BV contributed to the conceptualization of the review, fieldwork, draft write up, revisions and editing of the manuscript, provided leadership, and input to the review team at each stage of the project, contributed to the conceptualization, technical aspects, and critical revisions of the manuscript. Both authors contributed to the article and approved the submitted version.

## Funding

This work was supported by the South African Medical Research Council (SAMRC) under a Self-initiated Research Grant (No. SA4587ZA). ZO was supported by the National Research Foundation (No. 118160). Additional funding was received from the Oppenheimer Memorial Trust (No. OMT20829/02).

## Conflict of interest

The authors declare that the research was conducted in the absence of any commercial or financial relationships that could be construed as a potential conflict of interest.

## Publisher's note

All claims expressed in this article are solely those of the authors and do not necessarily represent those of their affiliated organizations, or those of the publisher, the editors and the reviewers. Any product that may be evaluated in this article, or claim that may be made by its manufacturer, is not guaranteed or endorsed by the publisher.

## Abbreviations

ALHIV: Adolescents living with HIV
ART: Antiretroviral therapy
SDGs: Sustainable Development Goals
SMoMW: Salutogenic Model of Mental Wellness
SOC: Sense of Coherence
UNICEF: United Nations Children's Fund
WHO: World Health Organization.
